# A single-centre, retrospective cohort study of paediatric tinea capitis in the United Kingdom

**DOI:** 10.1093/skinhd/vzag101

**Published:** 2026-07-10

**Authors:** Elliot Herd, Louis Gardner, Suzy Leech, Daniel Weiand, Anna Dubois, Luke Carson

**Affiliations:** School of Medicine, Newcastle University, Newcastle upon Tyne, UK; Department of Dermatology, Newcastle upon Tyne Hospitals NHS Foundation Trust, Newcastle upon Tyne, UK; Department of Dermatology, Newcastle upon Tyne Hospitals NHS Foundation Trust, Newcastle upon Tyne, UK; Department of Microbiology, Newcastle upon Tyne Hospitals NHS Foundation Trust, Newcastle upon Tyne, UK; Department of Dermatology, Newcastle upon Tyne Hospitals NHS Foundation Trust, Newcastle upon Tyne, UK; Department of Dermatology, Newcastle upon Tyne Hospitals NHS Foundation Trust, Newcastle upon Tyne, UK; Translational and Clinical Research Institute, Newcastle University, Newcastle upon Tyne, UK

## Abstract

**Background:**

Contemporary understanding of the epidemiology and clinical spectrum of paediatric tinea capitis in the UK is limited, with implications for both clinical management and public health interventions.

**Objectives:**

To examine recent trends in the incidence, clinical presentation and microbiological profiles of paediatric tinea capitis, and to explore the implications for quality of care and infection prevention.

**Methods:**

A single-centre retrospective cohort study was conducted of all patients aged < 18 years with microbiologically confirmed tinea capitis during two 3-year intervals: 2016–19 and 2022–25. Clinical records were reviewed for demographic features, clinical presentation, organisms identified and transmission sources, with a focus on practice standards and preventive strategies influencing quality of care.

**Results:**

A total of 102 patients were identified: 32 from 2016–19 and 70 from 2022–25, representing a 118.8% increase in cases. Severe inflammatory presentations (kerion) rose from 19% (*n* = 6/32) in 2016–19 to 36% (*n* = 25/70) in 2022–25. *Trichophyton tonsurans* remained the predominant organism in both cohorts (72% vs. 74% of cultured organisms), but *Trichophyton mentagrophytes* newly emerged as the second most common pathogen (9% in 2022–25), having been absent in 2016–19. Barber-associated transmission was only reported in 2022–25 (*n* = 22), exclusively among relatively older boys, who also had a higher rate of kerion (41%). A shift in preferred oral antifungal therapy from griseofulvin to terbinafine was observed with the changing microbiological profile.

**Conclusions:**

Our findings demonstrate a significant rise in paediatric tinea capitis, with a disproportionate increase in inflammatory cases and a shift in causative organisms. The emergence of barber-associated transmission and *T. mentagrophytes* highlights evolving risk factors and the need to adapt practice standards and preventive measures to influence quality of care. Enhanced surveillance targeted educational interventions for practitioners and public, and routine practice measures to moni­tor infection sources are warranted to improve the quality of care and patient outcomes.

## Teaser

There has been no recent large-scale UK study examining the incidence, severity, aetiology and microbiology of tinea capitis since 2003. Nevertheless, tinea capitis remains a prominent paediatric fungal infection, disproportionately affecting younger children, and carrying the risk of significant complications, including alopecia, permanent scarring and severe inflammatory lesions known as kerion. Although treatment relies on systemic antifungal agents, early recognition and preventative measures, such as improved hygiene practices and antifungal shampoos, may reduce disease burden. This single-centre retrospective cohort study provides new insight into the evolving epidemiology of tinea capitis in the UK. Clinical records of patients under 18 years of age with microbiologically confirmed infection treated at the Great North Children’s Hospital were analysed across two time periods, 2016–19 and 2022–25. Data on disease incidence, clinical severity, causative organisms and suspected transmission sources were compared in order to identify emerging trends. The study demonstrated a 118.8% increase in incidence between the two time periods, alongside a marked rise in kerion and a shift in causative organisms, suggesting increasing virulence. Notably, barber-associated transmission emerged as a significant suspected source. These findings highlight an urgent need for renewed public health awareness, targeted prevention strategies, and research into transmission dynamics and disease control.

What is already known about this topic?

- Previous UK studies, most recently in 2003, have described the incidence, risk factors and causative organisms of paediatric tinea capitis, but have not addressed recent trends or their implications for quality improvement.

What does this study add?

- This study identifies a rising incidence and severity of the capitis, a shift in microbiological causes and new evidence of ­barber-associated transmission.
- It highlights the importance of adapting clinical practice, surveillance and community education to these emerging trends.

Tinea capitis is a fungal infection of the scalp commonly seen in children. Clinical presentation includes enlarging pruritic, erythematous plaques, with alopecia. It may progress to scaling or develop a ‘black dot’ appearance due to hair shaft breakage at the scalp surface. Most cases of tinea capitis in the UK are caused by *Trichophyton tonsurans*.^1^ Globally, *Microsporum canis*, *Trichophyton violaceum* and *T. tonsurans* predominate.^2^ A more severe inflammatory variant, known as kerion, is characterized by boggy, tender swellings with pustules. Permanent scarring and alopecia may result. Kerion has been suggested to be predominantly caused by zoophilic organisms such as *Trichophyton verrucosum* and *Trichophyton mentagrophytes*.^3^ Cross-sectional data from a US study previously demonstrated an increased incidence in children with richly pigmented skin types.^4^

Griseofulvin remains the only licensed oral treatment option for children with tinea capitis in the UK, but it can be difficult to source. In clinical practice, oral terbinafine is commonly used off-license due to evidence of increased efficacy against *Trichophyton* species.^5^ Antifungal shampoos such as ketoconazole may be prescribed, primarily for preventative purposes rather than a definitive curative treatment.^6^ In the case of kerion or secondary bacterial infection, systemic antibiotics may also be used as an adjunct. Treating severe variants of tinea capitis early has been described to improve outcomes.^7^

The Great North Children’s Hospital in Newcastle upon Tyne, UK, provides tertiary paediatric dermatology care for North East England. In recent years, the clinical experience of the paediatric dermatology team has suggested a considerable increase in the number of referrals for tinea capitis. In addition, an increased severity of patient presentation, particularly kerion, has been noted. The impact of this on patients can be significant (Figure 1). Patients and parents often report an association with recent ­barber visits. Recent fashion changes favouring closely shaven hairstyles in boys and men may provide a hypothetical factor ­predisposing to infection. Barber-associated tinea capitis has ­previously been suggested in the literature, although in a small cohort, and without ­formal association with hairdressing practices.^8^

**Figure 1:**
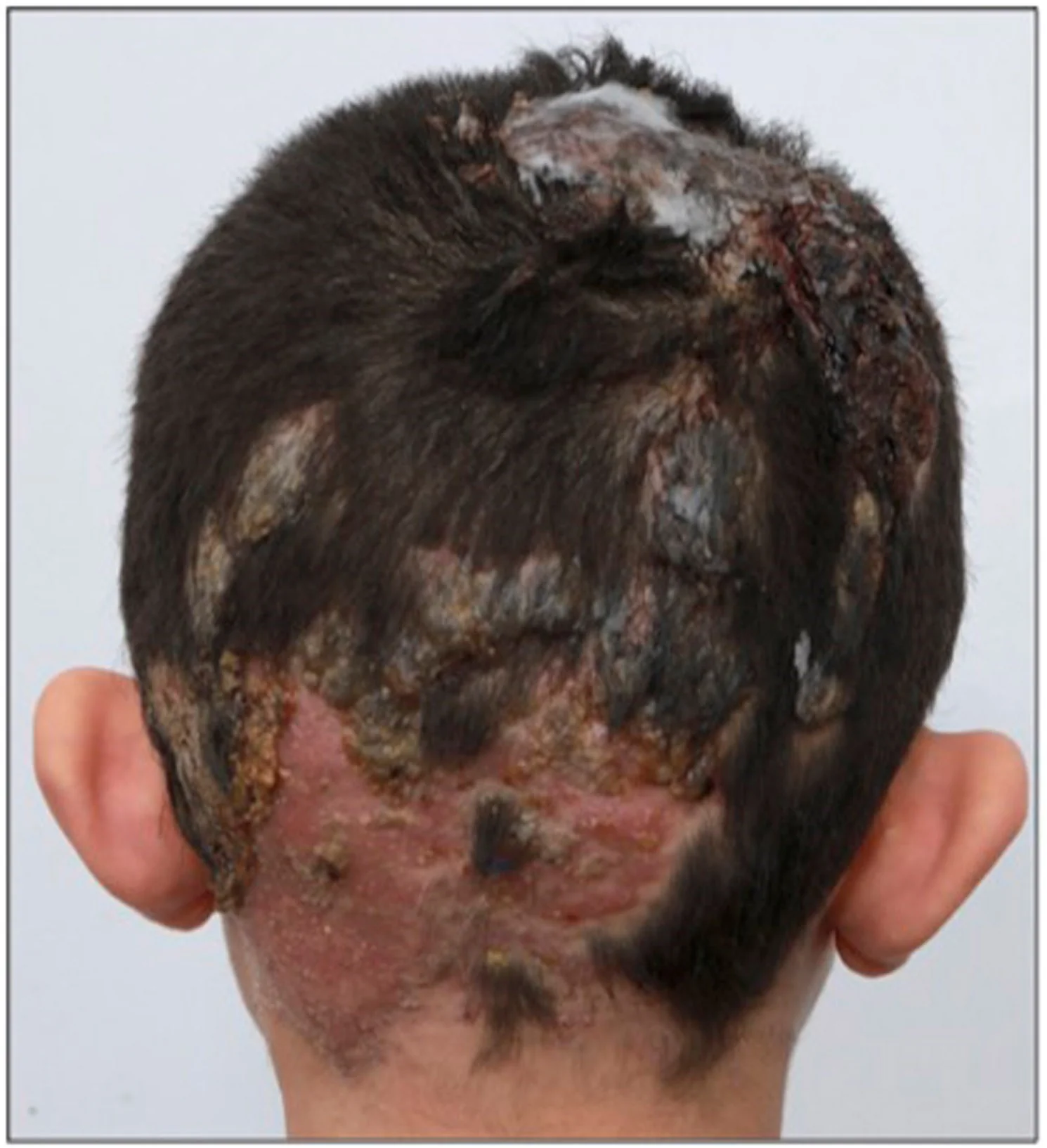
Clinical appearance of a severe fungal scalp infection.

The primary aim of our study was to formally compare the current incidence of culture-proven tinea capitis in the north east of England to a previous, time-matched cohort. Given the suspected increase in clinical severity, with more kerion-like phenotypes seen, we secondarily aimed to assess for a shift in causative pathogens. Other secondary aims included a description of patient-­suspected source of infection, and a description of the treatment of tinea capitis in a tertiary centre.

## Materials and methods

All positive mycology samples in patients aged < 18 years were identified via the Trust’s microbiology system across two 3-year periods: 2016–19 and 2022–25. The years 2020 to 2022 were excluded to minimize the influence of COVID-19 pandemic-related service disruptions, such as shifts in working practices and access to primary care. Clinical details on request forms were used to identify cases of tinea capitis and exclude cases of tinea corporis or onychomycosis. Potential cases were cross referenced with the National Data Opt-Out database and excluded if present.

Included cases were identified via National Health Service or hospital record number. These were subsequently used to access electronic health records, including dermatology clinic notes and primary care electronic records. Demographic data (age, sex, postcode deprivation decile), microscopy and culture results, clinical severity (described below), reported aetiology and treatment prescribed were extracted. Data on ethnicity could not be collected due to the unavailability of these data on included systems.

Kerion was identified through clinician documentation in dermatology notes. In cases where the term ‘kerion’ was not explicitly stated, diagnosis was inferred from clinical descriptions of tender, boggy scalp swellings. Any patient-reported or suspected sources of infection, such as pets, family members or recent haircuts or barber visits, were identified and recorded from dermatology and general practice notes.

Identification of dermatophyte infection and the causative organism was performed using microscopy and culture of skin scrapings and plucked hair samples. Specimens were collected and transported in commercially available packets designed ­specifically for the collection and transport of skin, nail and hair samples. Primary isolation of fungi was performed using two antibiotic-supplemented Sabouraud dextrose agar media ­inoculated in parallel: one containing both cycloheximide and ­chloramphenicol, and another containing chloramphenicol alone. Cycloheximide was included to inhibit the overgrowth of nondermatophyte moulds, while chloramphenicol served to prevent bacterial overgrowth. Where isolates could not be identified from primary culture, subculture was performed onto fresh chloramphenicol-supplemented Sabouraud dextrose agar, alongside malt agar to encourage sporulation. Cultures were incubated at 26–30 °C, as dermatophytes demonstrate suboptimal growth at temperature above 30 °C. Fungal identification was based on both the microscopic and macroscopic appearances. Methylene blue staining was used to examine fungal structures, including spores and hyphae. Colony morphology, growth rate, surface pigmentation and pigment production were also assessed. For example, *T. mentagrophytes* can be distinguished from *Trichophyton ­interdigitale* by its microscopic morphology of more spherical microconidia, and, typically, a greater number of macroconidia. In contrast, *T. tonsurans* is characterized by variable colony pigmentation, broad, irregular hyphae and the presence of clavate-to-pyriform microconidia (Figure 2).^9^

**Figure 2:**
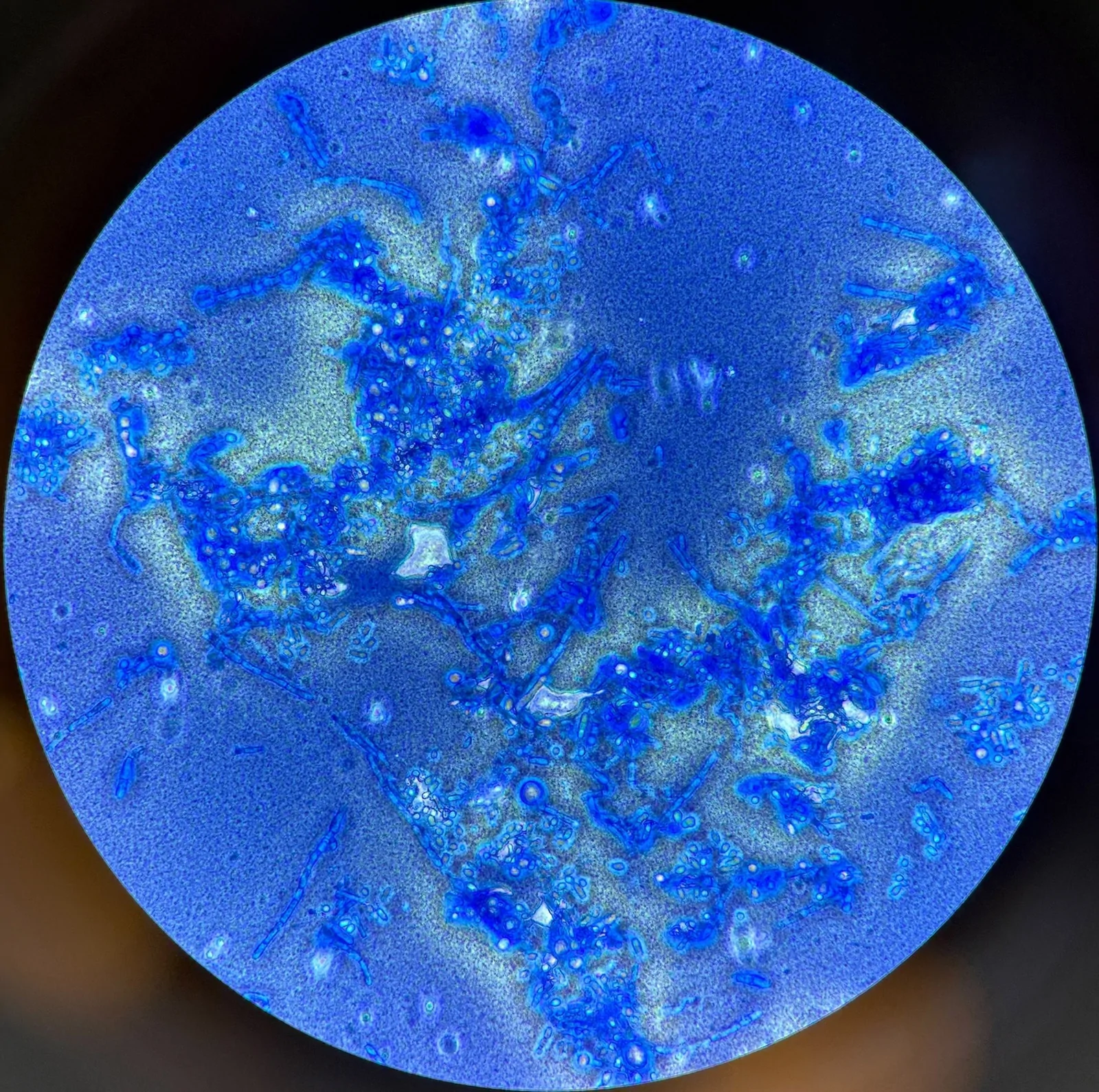
Methylene-blue stained examination of *Trichophyton tonsurans*, demonstrating septate hyphae, clavate-to-pyriform microconidia, and intercalary and terminal chlamydospores.

All laboratory work was conducted in a UKAS-accredited microbiology laboratory compliant with ISO 15189:2022 standards.^10^ The methods used were consistent with national guidance from the UK Health Security Agency UK Standards for Microbiology Investigations.^11^ Microscopy and culture results were obtained both from the initial laboratory records and from the patient notes. Cases with fungal hyphae seen on microscopy, but no fungal growth on culture, were excluded from microbiological trend analysis.

Treatment data included treatment prescribed in primary and secondary care. Treatment completion was defined by clinical resolution on examination by a dermatologist, as repeat ­mycological testing is not routinely performed unless there is a suspicion of disease recurrence. If the patient did not turn up for a further dermatologist appointment for this examination, treatment completion was also defined here. No changes in local treatment guidelines for tinea capitis occurred between 2016 and 2025. Data analysis was descriptive.

This study was reported in accordance with the STROBE reporting guideline.^12^ A completed STROBE checklist has been prepared and is available in the Supporting Information.

## Results

Data were collected across two 3-year time-periods: 17 June 2016 to 17 June 2019 (2016–19) and 17 June 2022 to 17 June 2025 (2022–25). Thirty-two patients had a positive mycology result from a hair sample or skin scraping from a tinea capitis infection in 2016–19 compared with 70 in 2022–25, identifying 102 patients overall for inclusion in the study. In 2016–19 there were 6 cases of kerion (of 32 total infections, 19%), compared with 25 cases in 2022–25 (of 70 total infections, 36%). The breakdown by year of infections and kerion phenotype is shown in Figure 3.

**Figure 3:**
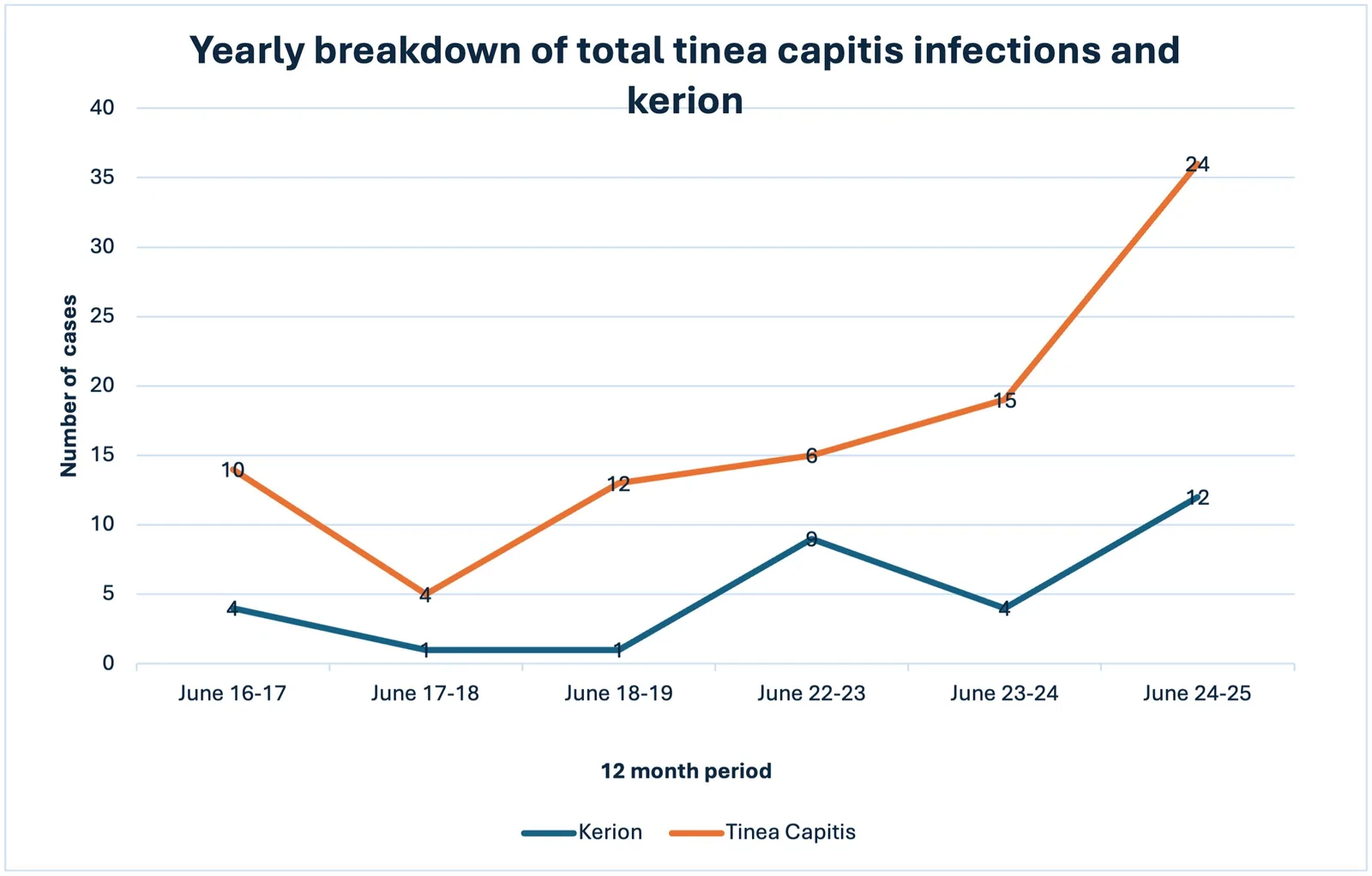
Annual distribution of tinea capitis and kerion cases.

Twenty-seven of the 32 suspected cases of tinea capitis from 2016–19 isolated a specific organism on fungal culture, with 5 cases positive for hyphae on microscopy, but with no growth on culture, or a non-tinea capitis isolated organism. Of these, 21 (78%) isolated *T. tonsurans* and 3 (11%) isolated ­*Trichophyton ­soudanense*. In 2022–25, 65 of the 70 cases isolated a specific organism. Of these, 49 were positive for *T. tonsurans* (75%) and 6 were positive for *T. mentagrophytes* (9%). Conversely, the incidence of *T. mentagrophytes* infections as a proportion of total infections rose, from 0% of total cases between 2016 and 2019 to 9.2% between 2022 and 2025. All *T. mentagrophytes* infections were seen from 2023 onwards. Conversely, the incidence of *T. soudanense* decreased, comprising 11% of total infections ­during 2016–19 compared with 1.5% from 2022–25. *Microsporum canis* was isolated only twice across the two cohorts, with both infections occurring between 2022 and 2025 (Table 1).

**Table 1:** The microbiological spectrum causing tinea capitis and kerion by year

Year	Organisms	Total cultures	Kerion cultures
2016–17	*Trichophyton tonsurans*	10	1
	*Trichophyton soudanense*	2	2
2017–18	*Trichophyton tonsurans*	2	0
	*Trichophyton interdigitale*	1	0
	*Trichophyton violaceum*	1	1
2018–19	*Trichophyton tonsurans*	9	1
	*Trichophyton rubrum*	1	0
	*Trichophyton soudanense*	1	0
Total 2016–19	*Trichophyton tonsurans*	21	2
	*Trichophyton soudanense*	3	2
	*Trichophyton interdigitale*	1	0
	*Trichophyton violaceum*	1	1
	*Trichophyton rubrum*	1	0
2022–23	*Trichophyton tonsurans*	6	3
	*Trichophyton rubrum*	2	1
	*Trichophyton soudanense*	1	0
	*Microsporum audouinii*	1	1
	*Trichophyton mentagrophytes*	1	1
	*Trichophyton interdigitale*	1	1
2023–24	*Trichophyton tonsurans*	11	3
	*Trichophyton mentagrophytes*	4	1
	*Microsporum audouinii*	2	0
	*Microsporum canis*	1	0
2024–25	*Trichophyton tonsurans*	32	10
	*Trichophyton mentagrophytes*	1	0
	*Microsporum canis*	1	0
	*Trichophyton violaceum*	1	0
Total 2022–25	*Trichophyton tonsurans*	49	16
	*Trichophyton rubrum*	2	1
	*Trichophyton soudanense*	1	0
	*Microsporum audouinii*	3	1
	*Trichophyton mentagrophytes*	6	2
	*Trichophyton interdigitale*	1	1
	*Microsporum canis*	2	0
	*Trichophyton violaceum*	1	0

Of the six cases of kerion from 2016–19, five had an identified organism on culture. Two (40%) of the infections were positive for *T. tonsurans*, two (40%) were positive for *T. soudanense* and one (20%) was positive for *T. violaceum*. Of the 25 kerion infections from 2022–25, 21 had an identified organism on culture. Sixteen (76%) were positive for *T. tonsurans* and two (10%) were positive for *T. mentagrophytes* (Table 1).

As a proportion of the 70 total *T. tonsurans* infections, 25.7% were associated with a kerion phenotype (9.5% in 2016–19 vs. 32.7% in 2022–25). In addition, 33.3% of T. mentagrophytes infections gave a kerion phenotype (exclusively isolated in 2022–25).

Patient demographics were also analysed (Table 2). Between 2016 and 2019, 20 of the 32 infections (63%) were found in male patients vs. 56 of 70 (80%) between 2022 and 2025. The mean (SD) patient age was 5.88 (2.68) years from 2016 to 2019 vs. 7.55 (4.13) years between 2022 and 2025. The mean deprivation decile of patients diagnosed with tinea capitis infection was 2.81 in 2016–19 compared with 3.41 in 2022–25.

**Table 2:** and treatment summary across both periods of study: 17 June 2016 to 17 June 2019 (2016–19) and 17 June 2022 to 17 June 2025 (2022–25)

Demographic and treatment data	2016–19 (*n* = 32)	2022–25 (*n* = 70)	Combined (*n* = 102)
Age (years), mean (SD)	5.88 (2.68)	7.55 (4.13)	7.03 (3.83)
Sex, *n* (%)			
Male	20 (63)	56 (80)	76 (74.5)
Female	12 (38)	14 (20)	26 (25.5)
Treatment (total prescriptions)			
Griseofulvin	16	3	19
Terbinafine	8	56	64
Itraconazole	5	8	13
Fluconazole	6	3	9
Topical antifungal monotherapy	2	5	7
Additional ketoconazole shampoo (with oral antifungal agent)	18	51	69
Treatment used for kerion and secondary bacterial infections (total prescriptions)			
Topical steroid	10	15	25
Flucloxacillin	5	13	18
Erythromycin	2	3	5
Co-amoxiclav	3	2	5
Clarithromycin	0	5	5
Treatment duration (weeks), mean (SD)			
Griseofulvin	7.81 (1.22)	6.67 (1.15)	7.63 (1.24)
Terbinafine	6 (3.16)	5.52 (2.00)	5.58 (2.18)
Itraconazole	5 (2.00)	6.75 (3.33)	6.07 (2.81)
Fluconazole	5.83 (2.71)	5 (2.12)	5.56 (2.28)

In 2016–19, no patients self-reported a suspected source of infection from barbers or recent visits to hairdressers compared with 22 of 70 patients in 2022–25. Nine of the 22 patients (41%) suspecting a haircut-associated infection were diagnosed with kerion. Of these 22 patients, 19 were culture-positive for a specific organism. Seventeen (89%) of these were positive for *T. tonsurans*, one (5%) positive for *T. mentagrophytes* and one (5%) positive for *Microsporum audouinii*. All 22 patients were boys [mean (SD) age 9.77 (4.13) years].

Household transmission was another patient-reported source of infection. Of the 102 patients, 28 (27%) reported recent exposure to an infected family member. Of these 28 patients, 24 yielded positive cultures for a specific organism. Among these, 17 (71%) were identified as *T. tonsurans*, 2 (8%) as *T. interdigitale*, 2 (8%) as *M. audouinii*, 1 (4%) as *M. canis* and 1 (4%) as *Trichophyton rubrum*. Four patients attributed their infection to household pets. Among these, *M. canis* was isolated in two cases (50%), *T. mentagrophytes* in one (25%) and *T. interdigitale* in one (25%). Dogs were mentioned as the pet species in two cases (involving *T. ­mentagrophytes* and *T. interdigitale*), whereas the specific animal species was not documented in either case that isolated *M. canis*. In the case ­associated with *T. mentagrophytes*, the same organism was also isolated from the family’s dog, and the patient subsequently developed kerion.

In 2016–19, 35 oral antifungal treatments were prescribed (Table 2), most commonly griseofulvin (*n* = 16), followed by terbinafine (*n* = 8), fluconazole (*n* = 6) and itraconazole (*n* = 5). This included a second-line antifungal prescription following failure of a first treatment, which was required for 6 of the 32 patients. Two patients received topical treatment only. Four of 32 (13%) patients had clinical recurrence following completed treatment. Two of these patients were initially prescribed griseofulvin: one initially prescribed terbinafine and one patient treated topically. A total of 13% (*n* = 2/16) of patients given griseofulvin and 50% (*n* = 1/2) of patients who received topical treatments alone had clinical recurrence. Of recurrent cases, the mean (SD) treatment duration with first-line oral antifungal medication prior to clinical relapse was 7 (4) weeks.

By comparison, in 2022–25, 70 oral antifungal treatments were prescribed (Table 2). The most prescribed antifungal treatment was terbinafine (*n* = 56), followed by itraconazole (*n* = 8), grise­o­fulvin (*n* = 3) and fluconazole (*n* = 3). This also included secondary antifungal prescriptions, which were given following failure of a first-line oral antifungal. A secondary antifungal was prescribed in 5 of the 70 patients (7%). All five of these patients were initially prescribed terbinafine. Five patients received topical treatment only, and one patient did not return for treatment. Seven of 70 (10%) patients had clinical recurrence, with 5 of these patients initially prescribed terbinafine and 2 receiving only topical treatments. A total of 9% of patients prescribed oral terbinafine and 40% of the patients receiving topical antifungals only had clinical recurrence.

Across both cohorts, 11 cases of disease recurrence were identified, of which an organism was successfully isolated in 8. All eight (100%) isolates were identified as *T. tonsurans*. Clinical recurrence following treatment was observed in 43% (*n* = 3/7) of patients who received topical treatment only compared with 9% (*n* = 6/64) of those treated with oral terbinafine and 11% (*n* = 2/19) of those treated with oral griseofulvin. The mean (SD) treatment duration for first-line oral antifungal medication in infections that subsequently recurred was 4 (3) weeks. The mean (SD) oral treatment duration in 2016–19 was 8.32 (3.83) weeks vs. 6.44 (3.49) weeks in 2022–25.

In 2016–19 there were 10 antibiotic prescriptions: 5 to treat kerion and 5 for secondary impetiginization. In 2022–25 there were 23 antibiotic prescriptions. Twenty of these were to treat kerion, with the remaining three prescribed for secondary impetiginization. Across both cohorts, flucloxacillin was the most prescribed antibiotic, prescribed 18 times (55% of all antibiotic prescriptions). Ketoconazole shampoo was prescribed alongside oral antifungal treatments 18 times in the 2016–19 cohort (56% of cases) vs. 51 times in 2022–25 (73% of cases).

## Discussion

This single-tertiary centre, retrospective cohort study demonstrated a 118.8% increase in the incidence of culture-proven tinea capitis among paediatric patients in 2022–25 compared with 2016–19. The largest rise was observed in 2024–25, with 36 ­culture-proven infections, exceeding the total of 32 culture-proven infections documented between 2016 and 2019.

*Trichophyton tonsurans* remained the most common pathogen, accounting for 78% of infections from 2016 to 2019 and 75% from 2022 to 2025. However, a pathogenic shift was noted in non-*T. ­tonsurans* infections. Notably, *T. mentagrophytes* was absent from all cases from 2016–19 but was isolated in six infections from 2022 to 2025. This species has become a subject of current concern, with genotype VII highlighted in a recent briefing by the UK Health Security Agency and an increasing number of cases being reported across Europe.^13,14^ A recent study in India also identified *T. mentagrophytes* as the most frequently isolated species among 153 cases of paediatric dermatophyte infections.^15^ The diversity of causative organisms also increased, from six species identified in 2016–19 to nine in 2022–25. While *T. violaceum* and *M. canis* are among the most prevalent species reported globally, each was isolated only twice across both cohorts, corroborating the prior suggestion that *T. tonsurans* predominates in the UK.^16^

The rise in incidence was accompanied by an increased proportion of severe cases, with a rise in the proportion of cases clinically, in keeping with kerion, from 19% in 2016–19 to 36% in 2022–25. Kerion presentations were mostly caused by *T. tonsurans*, with kerion representing 10% of total infections from this species from 2016–19 and 33% during 2022–25. The emergence of *T. mentagrophytes* as a causative pathogen in 2022–25 also caused two cases of kerion (33% of all *T. mentagrophytes* infections).

An increase in cases with a patient-reported suspected haircut-associated aetiology was also observed, comprising predominantly *T. tonsurans* infections (89%). This transmission route was identified exclusively among male patients and was associated with an older age profile, with a mean age of 9.77 years compared with the overall cohort mean age of 7.03 years. This demographic may be linked to shorter, closely cropped hairstyles, which could, hypothetically, facilitate an increased risk of transmission. This may, in part, have contributed to the observed rise in the incidence of tinea capitis between 2016–19 and 2022–25, a period that was likewise characterized by an increase in both mean patient age and the proportion of cases in male individuals. However, these findings should be interpreted with caution, as the suspected source was retrospectively collected and not consistently documented in clinical records and therefore may have been underestimated in both study periods. Household transmission represented 28% of infections, emphasizing the need for prompt and adequate treatment to prevent further spread, and further supporting the use of ketoconazole shampoo to minimize the risk of transmission.

The spectrum of prescribed antifungal medication changed over our study period. Griseofulvin was used to treat 50% of patients in 2016–19 but only 4% of patients in 2022–25 cohort. This change in practice is largely due to the decreased availability of griseofulvin, despite this being the only licensed treatment option. Terbinafine has become the most used oral antifungal medication, prescribed to 80% of patients in 2022–25 compared with 21% of patients in 2016–19. This change in choice of antifungal medication did not correlate with an increase in clinical recurrence rate. The mean number of weeks of treatment with oral antifungal medication decreased from 8.32 weeks in 2016–19 to 6.44 weeks in 2022–25 (Table 2). This was largely attributable to the shift in choice of oral antifungal medication from griseofulvin to terbinafine.

The clinical recurrence rate of tinea capitis was 4 out of 32 patients in 2016–19 (13%) and 7 out of 70 patients (10%) in 2022–25. The clinical recurrence rate was high where topical antifungal treatment was solely used (43%). These findings support current clinical practice, advocating for systemic antifungal treatment, with lower recurrence rates observed in patients treated with oral antifungal agents (9% for terbinafine, 11% for griseofulvin). Due to the recent treatment of patients at the time of analysis in the 2022–25 cohort, recurrence rates may be underestimated as short follow-up periods of the most recent cases may miss clinical recurrence.

This study reports a cohort of 102 patients over a period of 6 years, providing a considerable dataset for analysis and interpretation. This is the largest study of paediatric tinea capitis patients in the UK since 2003.^16^ Strict inclusion criteria with microbiologically confirmed disease facilitated focused data collection with potential relevance for clinical practice. As the first UK study, to our knowledge, of tinea capitis in children since the COVID-19 pandemic, it provides novel insights, including evidence of a pathogenic shift in the north east of England and a substantial increase in incidence. These findings have potential implications for public health awareness, management and quality-improvement strategies, particularly in populations at higher risk of adverse outcomes from the infection.

The retrospective study design imposes inherent limitations. Only patients with sufficient information in the electronic records were included, introducing the possibility of selection bias: cases of tinea capitis in individuals who did not attend primary care or dermatology services would not have been captured, potentially underestimating the true incidence. In addition, only cases with confirmed infection on microbiological testing (microscopy or culture) were included in our study, which has previously been shown to have poor sensitivity.^17^ Incorporating histological evidence of dermatophyte hair shaft invasion could have strengthened the diagnosis criteria. Nevertheless, the combination of ­specialist clinical demonstration of disease by a dermatologist, combined with positive skin scrapings was considered an acceptable diagnosis threshold. The original dataset includes patients diagnosed on microscopy alone, without organism isolation on subculture. However, subsequent analysis was limited to culture-confirmed cases only. Although these excluded cases represented a small proportion of the cohort (*n* = 10/102, 9.8% of all infections), this approach may have influenced the observed distribution of causative species. In our centre, clinical practice and guidelines relating to diagnosis of tinea capitis using skin and scalp scrapings have remained consistent throughout the study period, reducing the likelihood of bias introduced by changes in diagnostic approach. The single-centre nature of the study limits the generalizability of the findings to other settings or populations. Certain clinical details such as suspected sources of ­infection, including recent haircuts, were not consistently documented, limiting our data for this to descriptive, but defining a key area for further work and public health involvement. Further investigation is needed to determine whether the observed increase in paediatric tinea capitis represents a true rise in incidence or is instead driven by increased healthcare-seeking behaviour and referrals. However, as referral criteria remained unchanged across the study period, the latter explanation is less likely. Interpretation is further constrained by the scarcity of UK data on the impact of tinea capitis on children, with the last major study dating back to 2003.^16^

A consistent increase in the incidence of tinea capitis was shown among our paediatric population, with the most pronounced annual rise occurring during 2024–25. While recent trends for close shaven haircuts from barbers have been identified as a possible risk factor, this association could not be confirmed within the scope of the present study, due to recall bias that is inherent in retrospective self-reporting. The proportion of kerion increased disproportionately to the overall rise in ­incidence. A recent shift was identified in the distribution of non-*T. tonsurans* infections, including the emergence of *T. ­mentagrophytes*, which is of current public health concern. Further large-scale, multicentre studies are warranted to confirm these trends across broader populations.

## Supplementary Material

vzag101_Supplementary_Data

## Data Availability

The datasets analysed in this study contain patient-level information and are subject to strict confidentiality and information governance requirements. De-identified summary-level data may be made available upon reasonable request to the corresponding author. Requests should include a description of the proposed use and evidence of relevant research governance approval and will be evaluated against National Health Service information governance requirements and applicable data protection regulations.
